# Effect of extended photoperiod during winter on growth and onset of puberty in Murrah buffalo heifers

**DOI:** 10.14202/vetworld.2016.216-221

**Published:** 2016-02-27

**Authors:** Ashwani Kumar Roy, Mahendra Singh, Parveen Kumar, B. S. Bharath Kumar

**Affiliations:** Division of Dairy Cattle Physiology, National Dairy Research Institute, Karnal, Haryana, India

**Keywords:** buffalo, leptin, melatonin, metabolites, photoperiod, prolactin, puberty

## Abstract

**Aim:**

To investigate the effect of extended photoperiod on growth rate, hormonal levels, and puberty in Murrah heifers.

**Materials and Methods:**

About 14 Murrah buffalo heifers were divided into normal day photoperiod (NDP; n=7) and extended NDP (ENDP; n=7) groups. The ENDP group was exposed to 4 h of extended photoperiod with artificial light (160 lux) after sunset for 3 months during winter.

**Results:**

Group, age and group-by-age interaction effects on plasma glucose concentrations were non-significant (p>0.05). A significant effect of age on non-esterified fatty acids (p<0.05), cholesterol (p<0.01), and triglycerides (p<0.05) concentrations was observed. Group and group-by-age interaction effects on plasma T_3_, T_4_, leptin, 17 β estradiol, prolactin and melatonin concentrations were non-significant (p>0.05) while significant (p<0.05) age effect on T_4_, leptin and melatonin concentrations was observed. With respect to the circadian pattern of melatonin and prolactin, the group, time and group-by-time interaction effects were non-significant (p>0.05). Average daily gain and dry matter intake of heifers were non-significant between the NDP and ENDP groups but were comparatively higher in ENDP group. By the end of the experiment, 6 out of 7 heifers attained puberty in ENDP group in comparison to 4 out of 7 in NDP group.

**Conclusion:**

Extending the photoperiod by artificial light for 4 h during winter season resulted in better growth rate and early onset of puberty in Murrah buffalo heifers.

## Introduction

Buffaloes are the major source of milk production, and they contribute significantly to the economy of many countries in Southeast Asia [1]. More than 50% of the world population of 148 million buffaloes is reared in India. Late maturity, silent heat coupled with poor expression of estrus, irregular estrous cycle, seasonality in breeding, anoestrus, low conception rate, long postpartum interval, and repeat breeding are the well-known drawbacks leading to low productivity in this species [2-4]. Pubertal development involves physical, behavioral, and hormonal changes that are linked to the activation of the hypothalamic-adenohypophyseal-gonadal axis [5].

Management of photoperiod influences the attainment of puberty and prolactin secretion in beef heifers housed in an outdoor environment [6]. Onset of puberty in cattle is largely influenced by feed intake, quality of feed, and body weight (BW) gain [7]. Recent research has demonstrated that feeding replacement heifers to traditional target BW increased development costs without improving reproduction or subsequent calf production relative to development systems in which heifers were developed to lighter target BW ranging from 50% to 57% of mature BW [8,9]. Murrah buffaloes attain puberty between the ages of 33.1 and 36.5 months [10], whereas indigenous breeds such as Haryana, Kankrej and Sahiwal, reared under same management and environmental conditions, attain puberty at 24.6 months [11]. Feed efficiency improves by 9% in crossbred beef heifers by extending the photoperiod during winter [12]. Strong and clear estrous, increased progesterone, estradiol-17 β, and declined plasma melatonin in buffalo heifers exposed to 4 h of artificial light have been reported during autumn and winter seasons after sunset [13]. No significant changes in eating behavior, daily intake or live weight gain in buffalo heifers subjected to artificial light after sunset for 6 h have been found [14].

Although exposing the heifers to extended photoperiod seemed to be beneficial and economical, there are only a few investigations available in this regard indicating contradictory results. With this perspective, the present study was designed to investigate the effect of extended photoperiod on certain plasma hormones, metabolites, growth and onset of puberty in Murrah buffalo heifers during winter.

## Materials and Methods

### Ethical approval

The experiment was duly approved by the Institutional Animal Ethical Committee.

### Location and methodology

The experiment was conducted between the months of December and February at National Dairy Research Institute, Karnal, India, which is situated at an altitude of 250 m above mean sea level, latitude and longitude position being 29°42”N and 79°54”E, respectively. 14 Murrah buffalo heifers were selected and divided into control (n=7) and treatment (n=7) groups. A control group of heifers were exposed to natural photoperiod of 10.5 h. The treatment group heifers were exposed to 4 h of extended photoperiod with artificial light (160 Lux) after the sunset during the experimental period.

Daily feed intake and feed refusal of both the groups were recorded throughout the experiment. Dry matter intake (DMI) was calculated as the difference between feed intake and refusal. All the animals were reared under same management practices. BWs and blood samples were obtained from all the animals at fortnight intervals. To determine the circadian patterns of melatonin and prolactin hormones, blood samples were collected at an interval of 4 h over a period of 24-h. Immediately after collection, the samples were transported to the laboratory in an ice box, then centrifuged at 3000 rpm for 15 min to obtain plasma which was in different aliquots and stored at −20°C until analyzed for hormones and metabolites.

Plasma glucose was estimated by glucose oxidase-peroxidase method using commercial kits (Avecon Healthcare Pvt. Ltd.). Plasma cholesterol was estimated by cholesterol oxidase-phenol antipyrine (PAP) Trinder's method using commercial kits (Avecon Healthcare Pvt. Ltd.). Plasma triglycerides were estimated by glycerol phosphate oxidase-PAP Trinder's method using commercial kits (Avecon Healthcare Pvt. Ltd.). The copper soap solvent extraction method [15] was adopted for the estimation of plasma non-esterified fatty acids (NEFA). Progesterone (Cayman Chemical Company), estradiol-17 β, leptin, prolactin and melatonin (Cloud-clone Corp.) concentrations were estimated by enzyme immunoassays kits. The intra- and inter-assay coefficients of variation were <10% for all the hormones. Age at puberty was determined by behavioral signs, plasma progesterone levels [13] and ultrasound examination of the ovaries.

### Statistical analysis

Mixed model ANOVA (repeated measures linear model) was conducted to compare the BWs, metabolites, and hormone concentrations between normal day photoperiod (NDP) and extended NDP (ENDP) groups across the time periods. With respect to the circadian pattern of melatonin and prolactin, group, time and group-by-time interaction effects were determined by using mixed model ANOVA. The mean differences in BW, metabolites and hormone concentrations between the NDP and ENDP groups at each fortnight were analyzed by Student's t-test. GraphPad Prism (Version 5) and SPSS (Version 16) software was used to perform the statistical analysis.

## Results and Discussion

**Figure-1:**
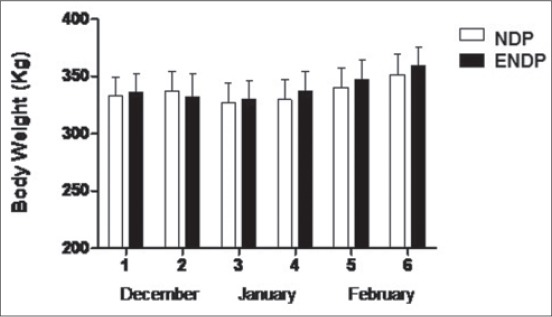
Mean (±standard error) body weight values in normal day photoperiod (NDP) and extended NDP groups.

**Figure-2:**
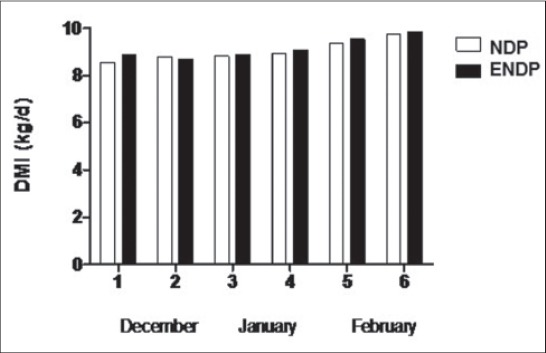
Mean (±standard error) dry matter intake in normal day photoperiod (NDP) and extended NDP groups.

BWs (Figure-1) and DMI (Figure-2) of heifers did not differ significantly (p>0.05) between short day (NDP) and extended short day photoperiods (ENDP). The mean ± standard error of mean (SEM) glucose concentrations in the short day (NDP) and extended short day photoperiod (ENDP) groups were 76.4±0.98 and 78.4±1.05 mg/dl, respectively (Table-1). There were no effects of group, age and group-by-age interaction on plasma glucose concentrations. The mean NEFA concentrations in NDP and ENDP groups were 136±53.6 and 168±54.6 mM/L, respectively. A significant (p<0.05) effect of age on NEFA concentrations was observed. A similar range of plasma glucose and NEFA was observed in Murrah buffaloes during winter [16]. The mean plasma cholesterol concentrations in NDP and ENDP groups were 92.3±5.75 and 93.8±4.72 mg/dl, respectively. There was significant (p<0.01) effect of age on plasma cholesterol concentrations. Plasma triglycerides concentrations in NDP and ENDP groups were 86.7±2.05 and 82.1±3.92 mg/dl, respectively. Significant (p<0.05) effect of age on triglycerides concentrations was observed.

**Table-1:** Mean±SE values of different metabolites in NDP and ENDP groups during the experimental period.

Fortnight	Glucose (mg/dl)		NEFA (μm/L)		Cholesterol (mg/dl)		Triglycerides (mg/dl)	
			
	NDP	ENDP	NDP	ENDP	NDP	ENDP	NDP	ENDP
1	77.2±1.54	75.8±3.21	40.0±16.2	159±128	101±7.19	98.0±5.90	91.0±4.21	74.3±5.40
2	79.41±2.61	80.67±2.97	363±144	413±111	110±13.1	108±6.97	86.3±7.52	84.7±10.1
3	77.26±2.33	81.37±4.59	158±66.7	98.9±41.1	101±5.94	102±7.24	78.6±8.77	70.0±3.90
4	76.71±2.10	76.17±2.63	190±78.3	132±47.9	77.6±3.47	88.9±5.28	87.8±8.60	97.5±5.11
5	75.39±1.60	76.35±2.10	40.3±18.84	186±139	79.7±6.25	76.9±6.10	83.8±4.67	85.0±4.57
6	72.24±1.51	80.20±2.20	24.1±4.25	18.0±8.64	82.5±5.91	87.5±12.5	92.4±4.91	81.0±3.84

NDP=Normal day photoperiod group, ENDP=Extended normal day photoperiod group, NEFA=Non-esterified fatty acids, SE=Standard error

**Figure-3:**
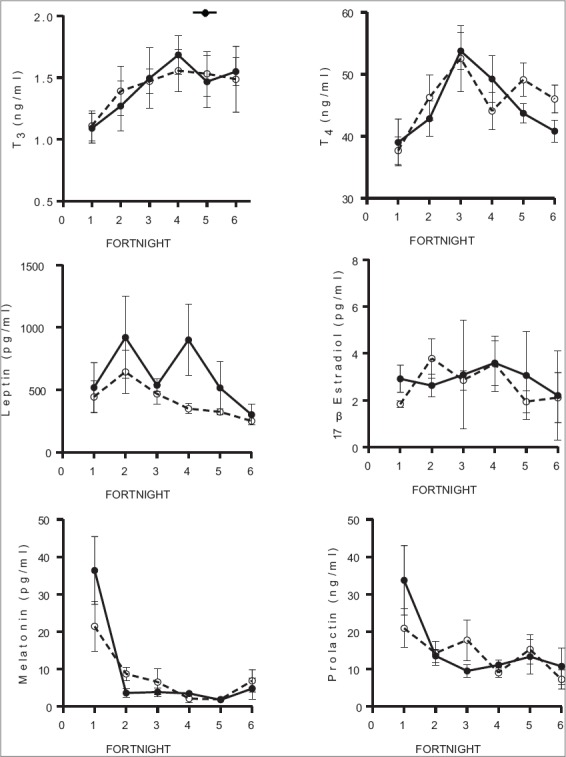
Mean (±standard error) plasma concentrations of T_3_, T_4_, leptin, 17 β estradiol, melatonin and prolactin in normal day and extended normal day photoperiod groups.

The mean ± SEM concentrations of plasma T_3_, T_4_, leptin, 17 β estradiol, prolactin and melatonin in NDP group were 1.43±0.07 ng/ml, 46.0±2.05 ng/ml, 413±56.4 pg/ml, 2.68±0.35 pg/ml, 14.1±2.11 ng/ml and 7.95±2.92 pg/ml, respectively (Figure-3). Plasma T_3_, T_4_, leptin, 17 β estradiol, prolactin and melatonin in ENDP group were 1.43±0.09 ng/ml, 44.9±2.27 ng/ml, 616±99.7 pg/ml, 2.92±0.19 pg/ml, 15.4±3.74 ng/ml, and 9.00±5.49 pg/ml, respectively. Group and group-by-age interaction effects on all the hormone concentrations were non-significant (p>0.05). Significant (p<0.05) age effect on plasma T_4_, leptin and melatonin concentrations was observed. The plasma T_3_ and T_4_ levels observed in this experiment was in accordance with a similar study conducted on peripubertal Murrah buffaloes [17]. The lower plasma 17 β estradiol concentrations (<5 pg/ml) observed in both the groups of this study was also in accordance with the results obtained [18]. By the end of the experiment, 6 out of 7 heifers attained puberty in ENDP group, whereas only 4 out of 7 heifers attained puberty in NDP group. Attainment of puberty was determined by behavioral signs, and confirmed by both plasma progesterone levels (>1 ng/ml) and presence of corpus luteum through ultrasound examination of ovaries.

**Figure-4:**
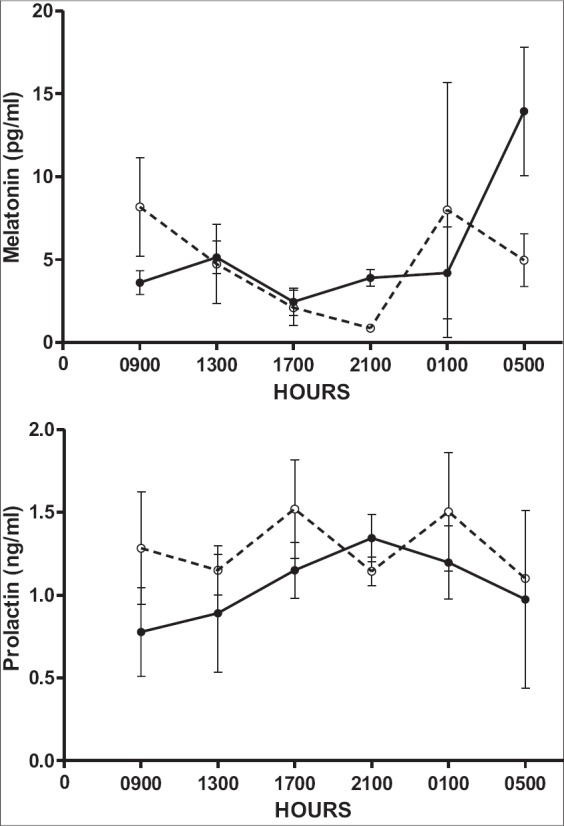
Circadian trend of prolactin and melatonin in normal day and extended normal day photoperiod groups.

The circadian pattern of melatonin and prolactin in both NDP and ENDP groups are depicted in Figure-4. There were no group, time and group-by-time interaction effects on both plasma melatonin and prolactin concentrations. Each heifer, irrespective of their group, showed different levels and pattern of melatonin and prolactin release. The plasma melatonin concentrations in NDP and ENDP groups ranged between 0.82-23.4 and 1.16-20.7 pg/ml, respectively. Plasma prolactin concentrations in NDP and ENDP groups ranged between 0.97-2.19 and 0.35-1.87 ng/ml, respectively. There was a definite circadian trend of plasma melatonin in buffaloes reared in few farms of Italy [19]. However, the buffaloes reared in a certain farm did not show a definite circadian pattern. The different trends of melatonin could be attributed to selection process practiced in the farms and targeted elimination of seasonal buffaloes [20]. The genetic selection criteria implemented to maintain the breeding herd in our farm reasons for the different pattern of melatonin release, irrespective of group.

In the present study, the DMI and BWs of NDP and ENDP groups did not differ significantly (p>0.05). However, the higher average daily gain was observed among the heifers of ENDP as compared to NDP group. Exposure of experimental group to 4 h of extended photoperiod with artificial light (160 Lux) after sunset may have increased their feed efficiency ratio and thus higher average daily gain. An increase in feed efficiency by extending photoperiod during winter in crossbred beef heifers corroborates the result of this study [21]. They also observed a non-significant difference in DMI between the natural and extended photoperiod groups, which agreed with our results. Plasma leptin appears to be an important link between metabolic status, the neuroendocrine axis and subsequent fertility in farm animals [21-23]. It also serves as a metabolic signal that acts on the hypothalamic-pituitary-ovarian axis to enhance gonadotropin-releasing hormone and luteinizing hormone secretion and ovarian function [24,25]. Proper management practices, nutrition, and optimum climatic conditions are indispensable for homeostasis and optimum productivity in cattle [26]. In the present study, the mean plasma leptin concentrations were comparatively higher in the ENDP (616±99.7 pg/ml) group than in NDP (413±56.4 pg/ml) group. An improved feed efficiency and better average daily gain in ENDP group may have influenced the plasma leptin concentrations and further attainment of puberty.

## Conclusion

Extending the natural photoperiod by artificial light (160 Lux) for 4 h daily during winter season resulted in better growth rate and early onset of puberty in Murrah buffalo heifers.

## Authors’ Contributions

AKR: Planning and execution of experiment. Drafted and revised the manuscript; MS: Hormone assays; PK: Management of animals in the farm; BSBK: Statistical analysis of data. All authors read and approved the final manuscript.
